# Adaptive and Mutational Responses to Peptide Dendrimer Antimicrobials in Pseudomonas aeruginosa

**DOI:** 10.1128/AAC.02040-19

**Published:** 2020-03-24

**Authors:** Fatma Ben Jeddou, Léna Falconnet, Alexandre Luscher, Thissa Siriwardena, Jean-Louis Reymond, Christian van Delden, Thilo Köhler

**Affiliations:** a Transplant Infectious Diseases Unit, University Hospitals Geneva, Geneva, Switzerland; b Department of Microbiology and Molecular Medicine, University of Geneva, Geneva, Switzerland; c Department of Chemistry and Biochemistry, University of Bern, Bern, Switzerland

**Keywords:** *Pseudomonas aeruginosa*, AMP-dendrimers, two-component systems, antimicrobial peptides, dendrimers, two-component regulatory systems

## Abstract

Colistin (polymyxin E) is a last-resort antibiotic against multidrug-resistant isolates of Pseudomonas aeruginosa. However, the nephro-toxicity of colistin limits its use, spurring the interest in novel antimicrobial peptides (AMP). Here, we show that the synthetic AMP-dendrimer G3KL (MW 4,531.38 Da, 15 positive charges, MIC = 8 mg/liter) showed faster killing than polymyxin B (Pmx-B) with no detectable resistance selection in P. aeruginosa strain PA14. Spontaneous mutants selected on Pmx-B, harboring loss of function mutations in the PhoQ sensor kinase gene, showed increased Pmx-B MICs and *arnB* operon expression (4-amino-l-arabinose addition to lipid A), but remained susceptible to dendrimers. Two mutants carrying a missense mutation in the periplasmic loop of the PmrB sensor kinase showed increased MICs for Pmx-B (8-fold) and G3KL (4-fold) but not for the dendrimer T7 (MW 4,885.64 Da, 16 positive charges, MIC = 8 mg/liter). The *pmrB* mutants showed increased expression of the *arnB* operon as well as of the *speD2-speE2*-PA4775 operon, located upstream of *pmrAB*, and involved in polyamine biosynthesis. Exogenous supplementation with the polyamines spermine and norspermine increased G3KL and T7 MICs in a *phoQ* mutant background but not in the PA14 wild type. This suggests that both addition of 4-amino-l-arabinose and secretion of polyamines are required to reduce susceptibility to dendrimers, probably neutralizing the negative charges present on the lipid A and the 2-keto-3-deoxyoctulosonic acid (KDO) sugars of the lipopolysaccharide (LPS), respectively. We further show by transcriptome analysis that the dendrimers G3KL and T7 induce adaptive responses through the CprRS two-component system in PA14.

## Abstract (precis)

Colistin (polymyxin E) is a last-resort antibiotic against multidrug-resistant isolates of Pseudomonas aeruginosa. However, the nephro-toxicity of colistin limits its use, spurring the interest in novel antimicrobial peptides (AMP). Here, we show that the synthetic AMP-dendrimer G3KL (MW 4,531.38 Da, 15 positive charges, MIC = 8 mg/liter) showed faster killing than polymyxin B (Pmx-B) with no detectable resistance selection in P. aeruginosa strain PA14.

## INTRODUCTION

Antimicrobial peptides (AMP) represent the most diverse class of antibiotics. They are synthesized by animals, plants, and microorganisms and show a broad spectrum of activity (1, 2). Their chemical structure includes linear peptides like LL-37, a human AMP (3), or cyclic peptides like polymyxins produced by the bacterium Paenibacillus polymyxa (4). The positively charged polymyxins interact with the negatively charged phosphate residues of the lipid A, thereby displacing the Mg^2+^ ions that stabilize the lipopolysaccharide (LPS) in the outer membrane of Gram-negative bacteria (5–7). Subsequent insertion of the polymyxins into the lipid layer causes phospholipid perturbations in both membranes, leading to osmotic imbalance and cell death. Nonlytic AMPs may also have intracellular targets and can block cell division by different mechanisms (8, 9). The most widely used AMP in the clinical setting is colistin (polymyxin E), which has gained renewed interest because it is efficacious against multidrug-resistant (MDR) Gram-negative bacteria (10–12).

However, due to the toxicity of polymyxins (13, 14) and the emergence of resistance against colistin in clinical settings, alternative AMPs are warranted (15–17). We recently reported the synthesis of a group of dendrimer AMPs, characterized by a synthetic branched-chain peptide scaffold consisting exclusively of D-Leu and D-Arg amino acid residues (18–20). The dendrimer molecules tested here are characterized by MWs of 4,500 to 5,000 Da and present 15 to 17 positive charges at neutral pH. G3KL is a representative of these third-generation dendrimers, which shows activity against MDR Pseudomonas aeruginosa clinical isolates as well as Acinetobacter baumannii and *Enterobacteriaceae* harboring extended-spectrum β-lactamases (ESBLs) (21, 22). G3KL has low hemolytic activity, with minimal hemolytic concentrations above 2,000 μg/ml (21) and IC_50_ values around 1,000 μg/ml for HeLa and CHO cells (23). G3KL and its derivatives are synthesized with D-amino acids conferring increased stability against proteolytic cleavage in the presence of human serum (21). Furthermore, G3KL was shown to have an angiogenic effect on injured human fibroblast cells (24). G3KL-like dendrimers are currently under preclinical development.

Since the physicochemical properties of the synthetic dendrimer antimicrobial peptides differ substantially from natural AMPs like polymyxins, we wondered whether dendrimers and polymyxins were affected by similar resistance mechanisms. Surprisingly, attempts to select spontaneous mutants with G3KL were unsuccessful; however, mutants selected on Pmx-B, harboring specific mutations in the PmrB sensor kinase, showed decreased susceptibility to both G3KL and Pmx-B. We characterized these mutants and found that two different mechanisms affecting the surface charge of the LPS are required to achieve decreased susceptibility to G3KL.

## RESULTS

### Dendrimers have a low spontaneous resistance emergence frequency.

**FIG 1:**
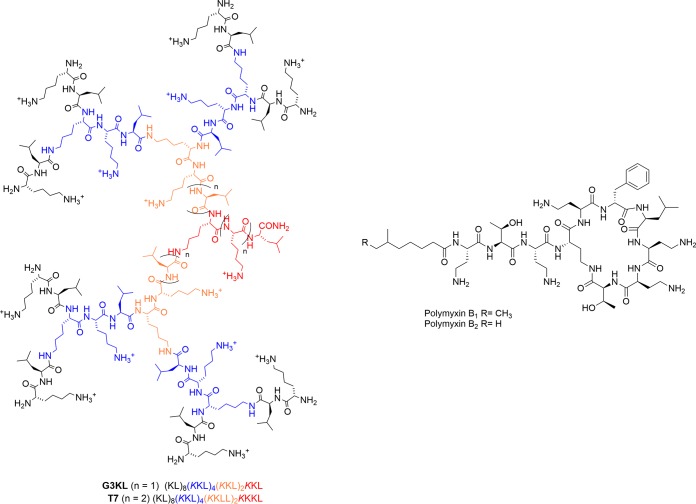
Chemical structures of dendrimers G3KL and T7 and polymyxin B. Dendrimers G3KL (MW 4,531.38 Da, 15 positive charges) and T7 (MW 4,885.64 Da, 16 positive charges) are representatives of third-generation AMP dendrimers. First, second, and third generation residues are indicated in orange, blue, and black color, respectively. Polymyxin B was used as comparator (MW 1,301 Da, 5 positive charges).

G3KL and T7 are representatives of third-generation dendrimer molecules (21). They differ in their amino acid composition and molecular weight but have similar numbers of positive charges (Fig. 1) and are active against P. aeruginosa reference strains and MDR clinical isolates (MIC = 8 mg/liter) (19). To evaluate the possibility of resistance emergence to dendrimer AMPs, we attempted to select spontaneous mutants with P. aeruginosa strain PA14 on agar plates using polymyxin B (Pmx-B) as a comparator (Fig. 1). While Pmx-R mutants appeared on Mueller-Hinton broth (MHB) agar plates at a frequency of 10^−7^ to 10^−8^ on 2× or 4× the MIC, no colonies could be obtained at 2× or 4× the MIC with G3KL or T7 even after prolonged incubation (72 h). We thus performed a selection in liquid medium in a microtiter plate format, exposing PA14 to increasing G3KL and T7 concentrations. After six serial passages, we were still unable to obtain a stable mutant showing increased MICs to G3KL, although such mutants emerged readily with Pmx-B. However, one mutant colony (2P4) was obtained after six passages with the dendrimer T7.

To investigate a potential cross-resistance between the different AMPs, we tested colonies obtained during the initial Pmx-B selection on agar plates for their susceptibilities to G3KL and T7. Among seventeen randomly selected colonies from the Pmx-B-supplemented plates (4× the MIC of Pmx-B), fifteen were resistant to Pmx-B alone (4.1 to 4.17) and only two colonies (4.18 and 4.19) showed increased MICs for both Pmx-B and G3KL (Table S1 in the supplemental material). The single colony selected on T7 showed decreased susceptibility to Pmx-B and to the other dendrimer G3KL (Table 1). Hence, Pmx-B can select for mutants with decreased susceptibility to G3KL. However, the dendrimers showed an extremely low resistance selection frequency.

**TABLE 1:** Susceptibilities and mutations of AMP-resistant mutants

Strain	Selection	MIC (mg/liter)			Mutated gene	Amino acid modification
		Pmx-B	G3KL	T7		
PA14	none	1	8	8	wild-type	none
4.13	Pmx-B	8	8	8	*phoQ*	Val369fs^*a*^
4.18	Pmx-B	8	32	16	*pmrB*	Thr132Pro^*a*^
4.19	Pmx-B	8	32	16	*pmrB*	Phe124Cys
2P4	T7	16	64	32	*pmrB*	Asp47Glu^*a*^

a Mutations determined by whole-genome sequencing.

### Cross-resistance between Pmx-B and dendrimers results from alterations in the PmrB sensor kinase.

**FIG 2:**
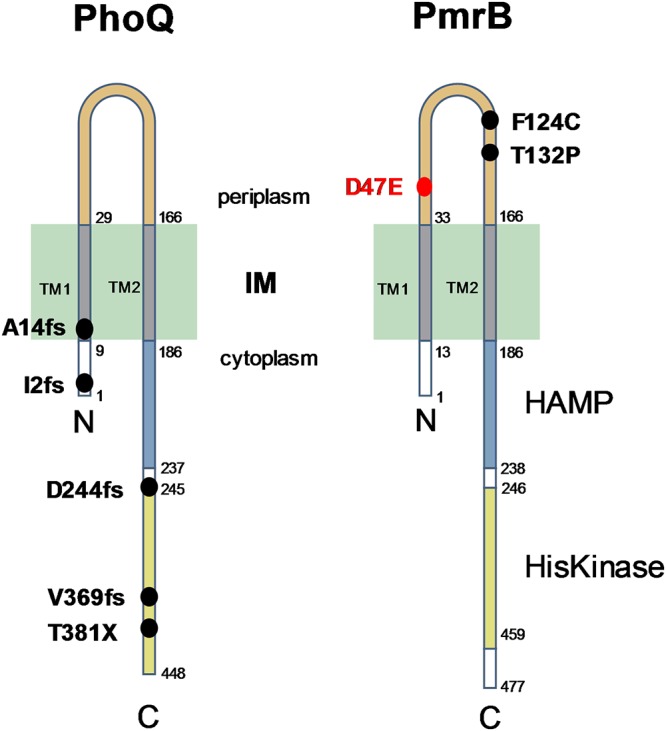
Mutations selected in the TCS sensor kinases PhoQ and PmrB. Mutations selected by Pmx-B are shown by black dots, while the only mutation selected by T7 is shown in red (Table S1). Mutations occurring in PhoQ are exclusively nonsense or frameshift mutations, leading to nonfunctional PhoQ variants. These confer resistance to Pmx-B only. In contrast, mutations selected in PmrB are missense mutations located in the periplasmic loop. These confer decreased susceptibility to Pmx-B and G3KL and are likely gain-of-function mutations. The HAMP and histidine kinase domains are highlighted in blue and yellow, respectively. TM, transmembrane segment; IM, inner membrane. Membrane topology predictions were generated using OCTOPUS (http://octopus.cbr.su.se/).

One colony from each of the three resistance profiles (4.13, 4.18, and 2P4) was submitted to whole-genome sequencing. Mutant 4.13, which is resistant only to Pmx-B (MIC = 8 mg/liter), showed a 4-nucleotide (nt) insertion at position 1,105, resulting in a frameshift mutation at codon Val369 of PhoQ, a two-component system (TCS) sensor kinase frequently mutated in colistin-resistant clinical isolates (Table 1 and Fig. 2) (25). The cross-resistant mutant 4.18, also selected on Pmx-B, showed a single nucleotide change in the *pmrB* gene, encoding another TCS sensor kinase (26). This mutation resulted in a Thr-to-Pro substitution at position 132, located in the periplasmic domain of PmrB (Fig. 2). The mutant obtained with the dendrimer T7 carried a single nucleotide change in the *pmrB* gene, resulting in an Asp-to-Glu substitution at position 47, also located in the periplasmic loop of PmrB (Table 1 and Fig. 2). Based on these results, we sequenced the *phoQ* and *pmrB* genes in the remaining spontaneous mutants selected on Pmx-B. Mutant colonies resistant only to Pmx-B showed alterations in the *phoQ* gene resulting from deletions or short nucleotide insertions (Table S1). The remaining colony displaying cross-resistance to Pmx-B and G3KL (4.19) carried a single nt change in *pmrB*, resulting in a Phe-to-Cys substitution at position 124, located again in the periplasmic loop of PmrB (Table 1 and Fig. 2). We conclude that frameshift mutations inactivating the PhoQ sensor kinase conferred resistance to Pmx-B only, while specific amino acid substitutions occurring in the periplasmic loop of the sensor kinase PmrB were required to confer decreased susceptibility to Pmx-B and the two dendrimers.

### G3KL preincubation protects against Pmx-B and dendrimer killing.

**FIG 3:**
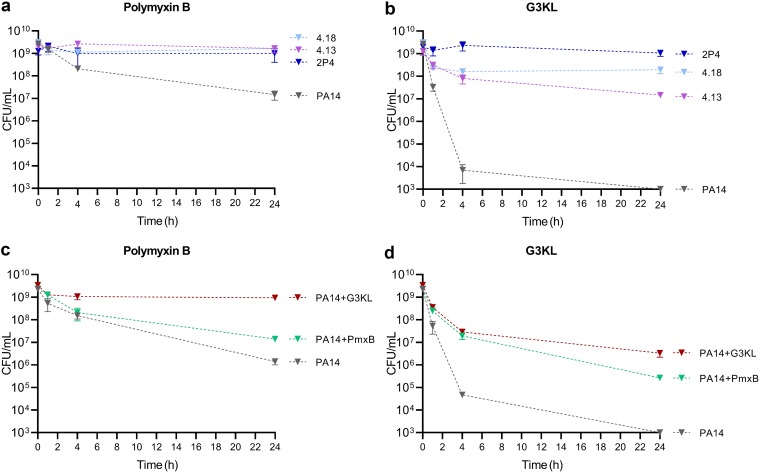
Killing challenge by Pmx-B and G3KL against wild-type PA14, the phoQ mutant 4.13, and the pmrB mutants 4.18 and 2P4. Killing assays were performed with bacterial cell suspensions in M9 medium supplemented with 2 mM MgSO_4_ and 6× the MIC of either Pmx-B (6 mg/liter) or G3KL (48 mg/liter). (a) The *phoQ* and *pmrB* mutants showed similar survival when challenged with Pmx-B. (b) In contrast, the *pmrB* mutants showed increased survival compared to the *phoQ* mutant when challenged with G3KL. (c and d) Preincubation with either Pmx-B (c) or G3KL (d) increased the fraction of surviving cells upon subsequent exposure to 6× the MIC of Pmx-B or G3KL. Killing curves were performed in technical triplicates and repeated on three separate occasions. Values are the mean and standard deviations from one representative experiment.

To compare the killing kinetics between dendrimers and Pmx-B, we performed time-kill assays with the PA14 wild-type strain, the *phoQ* mutant 4.13, and the two *pmrB* mutants 4.18 and 2P4 at 6× the MIC of either Pmx-B or G3KL. Pmx-B caused a 2-log reduction in CFU after 24 h for the wild-type, while CFU of the *phoQ* and the two *pmrB* mutants remained unaffected (Fig. 3a). In comparison, G3KL caused a faster killing than Pmx-B of PA14 cells, resulting in a final 6-log reduction of viable counts at 24 h. The *phoQ* mutant 4.13 showed a 2-log reduction in CFU after 24 h, while the CFU of the *pmrB* mutants 4.18 and 2P4 remained stable during 24 h (Fig. 3b).

To investigate, whether G3KL-mediated killing was affected by prior exposure to AMPs, we precultured the PA14 wild type for 16 h in the presence of Pmx-B or G3KL at 0.25× the MIC. Preincubation by Pmx-B increased survival by 1 log or 2 log when challenged subsequently with 6× the MIC of Pmx-B or G3KL, respectively (Fig. 3c). In comparison, preincubation with G3KL increased the proportion of surviving PA14 cells by 3 log or 4 log when challenged with Pmx-B or G3KL, respectively (Fig. 3d). The cross-protection effect between Pmx-B and G3KL suggests the involvement of common adaptive resistance mechanisms for both AMPs.

### Role of lipid A modifications for Pmx-B and G3KL activity.

**FIG 4:**
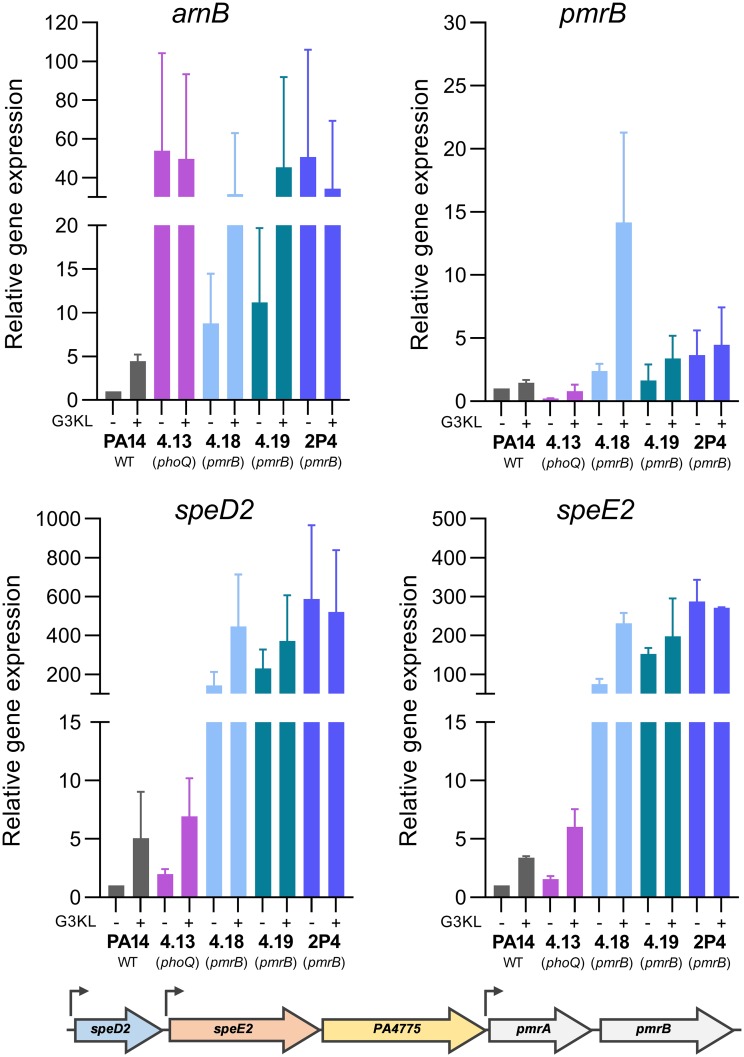
Expression analysis of *arnB*, *pmrB*, *speD2*, and *speE2*. genes by qPCR. Prior to RNA extraction, bacterial strains were grown for 18 h in the presence or absence of 0.25× MIC of G3KL in MHB medium in microtiter plates. Fold changes are calculated relative to PA14 without G3KL exposure. Results are the mean and standard deviations of two independently performed experiments. The operon structure of the *speD2* operon and putative promoters (arrows) are shown below.

PhoQ is the main target for mutations leading to polymyxin resistance in P. aeruginosa clinical isolates (25). The PhoPQ TCS regulates, among others, the expression of the *arnBCADTEF* operon responsible for the synthesis of 4-amino-4-deoxy-l-arabinose (l-Ara4N) and its addition to the lipid A sugar portion of the LPS (27). We therefore measured the expression of the *arnB* gene in PA14 and in the selected mutants 4.13, 4.18, 4.19, and 2P4 in the presence and the absence of G3KL. As shown in Fig. 4a, expression of *arnB* was increased 17-fold compared to PA14 in the *phoQ* mutant 4.13, while in the *pmrB* cross-resistant mutants (4.18, 4.19, and 2P4) *arnB* levels were increased 4- to 12-fold. The expression of *arnB* was induced in the presence of 0.25× MIC of G3KL in the wild-type strain as well as in the two cross-resistant mutants 4.18 and 4.19, but not in mutant 2P4. This suggests that modification by the *arn* operon could affect susceptibility to G3KL and T7. We therefore constructed a deletion in the *arnT* gene, encoding the l-Ara4N transferase. In the PA14 wild type this resulted in a weak but reproducible 2-fold decrease in G3KL MICs, without affecting Pmx-B MICs (Table 2). Inactivation of *arnT* in the *phoQ* mutant 4.13 resulted in a 4-fold decrease in Pmx-B MICs but had no effect on G3KL MICs. Finally, when *arnT* was deleted in the cross-resistant mutant 4.18, MICs for both AMPs decreased 4-fold (Table 2). These data suggest that addition of l-Ara4N to the LPS affects susceptibility to G3KL. However, the expression level of the *arn* operon did not correlate with the G3KL MICs, since in the *phoQ* mutant 4.13, which showed the highest *arnB* expression, G3KL MICs were unaffected (Table 2). We therefore suspected that additional mechanisms might be required for decreased dendrimer susceptibility.

**TABLE 2:** Effect of *arnT* deletion on Pmx-B and G3KL susceptibilities

Strain	MIC (mg/liter)		*arnB* expression^*a*^
	Pmx-B	G3KL	
PA14	1	8	low
PA14 Δ*arnT*	1	4	ND
4.13 *phoQ*	8	8	high
4.13 *phoQ*Δ*arnT*	2	8	ND
4.18 *pmrB*	8	32	intermediate
4.18 *pmrB*Δ*arnT*	2	8	ND

a ND, not determined.

### Involvement of operon *speD2-speE2*-PA4775 in G3KL susceptibility.

Since the cross-resistant mutants carried mutations in the *pmrB* gene, we decided to measure gene expression of the *pmrAB* operon as well as of the upstream genes *speD2-speE2*-PA4775 (referred to as the *speD2* operon), which have been reported to be regulated by the PmrAB TCS (28). As expected, the expression of *pmrB* was increased in the *pmrB* mutants 4.18, 4.19, and 2P4, suggesting a positive autoregulation loop, while in the *phoQ* mutant 4.13, expression of *pmrB* was comparable to the PA14 wild-type strain (Fig. 4b). G3KL caused a 5-fold induction of *pmrB* expression in mutant 4.18 bearing a mutation in the periplasmic loop of PmrB. Expression of *speD2* and *speE2* showed at least a 100-fold increase in the *pmrB* mutants, while in the *phoQ* mutant the expression was comparable to that in PA14 (Fig. 4c and d). Preincubation of the strains with subinhibitory concentrations of G3KL induced expression of *speD2* and *speE2* in PA14, the *phoQ* mutant 4.13, and the *pmrB* mutant 4.18, but not in the *pmrB* mutants 4.19 and 2P4. This result strongly suggested an implication of the *speD2* operon in decreased susceptibility to dendrimers.

### Overexpression of polyamine synthesis operon increases G3KL resistance but only in the *phoQ* mutant.

The *speD2* operon was previously shown to be involved in the synthesis of polyamines (28, 29). We thus postulated that polyamines might play a role in the decreased susceptibility of the *pmrB* mutants to G3KL. We therefore cloned and expressed the three genes of this operon individually or in combination and evaluated the effect on the activity of G3KL and Pmx-B. Plasmid-mediated expression of the individual genes or pair of genes had no effect on G3KL MICs. However, we observed an increase in G3KL MICs when the three genes were coexpressed in the *phoQ* mutant 4.13 (>4-fold increase in MICs), but not in the wild-type PA14 (Table 3 and Table S2). In contrast, T7 MICs showed only a 2-fold increase when the three genes were coexpressed in the *phoQ* mutant 4.13, suggesting that dendrimers do not have a uniform behavior in response to LPS modifications. To confirm the role of the *speD2* operon, we deleted by homologous recombination the *speE2* gene in the *pmrB* mutants 4.18 and 4.19. Indeed the MICs of G3KL decreased for the 4.18 Δ*speE2* and 4.19 Δ*speE2* mutants relative to the PA14 wild-type strain, while the MICs of Pmx-B were not affected (Table 3). This clearly demonstrates the specific role of the *speD2* operon in the decreased susceptibility to dendrimers in the cross-resistant mutants 4.18 and 4.19, and suggests that polyamine synthesis is an essential determinant for the increased G3KL MICs, but requires prior modification of lipid A by addition of l-Ara4N.

**TABLE 3:** Effect of *speD2-speE2*-PA4775 operon on AMP susceptibility

Strain	MIC (mg/liter)		
	Pmx-B	G3KL	T7
PA14	1	8	8
PA14 pIApX2 (vector)	1	8	8
PA14 pspDE2-5	1	16	8
4.13	8	16	8
4.13 pIApX2 (vector)	16	8	8
4.13 pspDE2-5	8	**>64**^*a*^	16
4.18	8	64	16
4.18 *ΔspeE2*	8	8	8
4.19	8	64	16
4.19 *ΔspeE2*	8	8	8

a Value in bold indicates > 4-fold MIC increase compared to vector control.

### Externally added polyamines affect G3KL activity.

P. aeruginosa and most Gram-negative bacteria produce cadaverine, putrescine, and spermidine as the major polyamines (30). The polyamine synthesized by the *speD2* operon has been suggested recently to correspond to norspermidine (29). We therefore tested several commercially available polyamines displaying various positively charged amines and carbon chain lengths. We found that spermidine (C_7_H_19_N_3_) and norspermidine (C_6_H_17_N_3_), both carrying three amine groups, had no effect on AMP MICs. Instead, we observed that spermidine increased susceptibility to Pmx-B and G3KL by 2- to 4-fold, but only for the *pmrB* mutants (underlined values in Table 4). In contrast, spermine (C_10_H_26_N_4_) and norspermine (C_9_H_24_N_4_), both harboring four amine groups, increased MIC values of Pmx-B and G3KL in the *phoQ* mutant 4.13 but not in the wild type (bold values in Table 4). Norspermine also increased G3KL MICs in two of the three *pmrB* mutants (Table 4). These results are in agreement with the hypothesis that endogenously synthesized polyamines are required for decreased susceptibility to dendrimers.

**TABLE 4:** Effect of externally added polyamines on AMP activities

Strain	Mutation	MIC (mg/liter) ^*a*^^,^^*b*^									
		Pmx-B in presence of:					G3KL in presence of:				
		No polyamine	SpD	nSpD	SpN	nSpN	No polyamine	SpD	nSpD	SpN	nSpN
PA14	WT	1	<0.5	1	1	1	8	4	8	16	16
4.13	*phoQ*	8	8	16	**32**	**32**	8	16	8	**>64**	**>64**
4.18	*pmrB*	8	2	8	4	8	32	8	32	32	**>64**
4.19	*pmrB*	8	2	8	4	8	32	8	64	32	**>64**
2P4	*pmrB*	16	8	8	8	8	>64	16	>64	32	>64

a SpD, spermidine; nSpD, norspermidine; SpN, spermine; nSpN, norspermine (added at final conc. of 1 mM).

b MIC values in bold indicate at least a four-fold decrease in susceptibility; underlined MIC values indicate at least a four-fold increase in susceptibility.

### Dendrimers induce *arn* and *speD2* operon expression.

**FIG 5:**
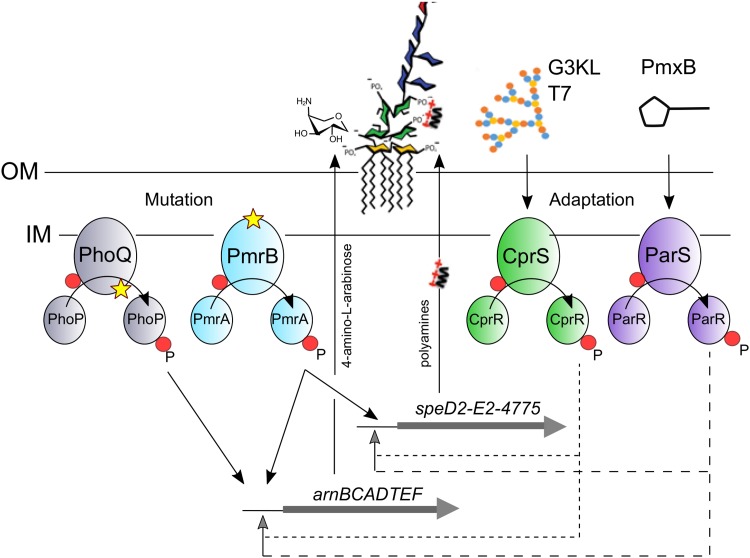
Simplified scheme for the response of P. aeruginosa PA14 to G3KL and Pmx-B. Mutations leading to increased Pmx-B MICs occurred either in PhoQ (loss-of-function mutations) or Pmr-B (likely gain-of-function mutations), indicated by the star symbol. PhoQ mutations only affected *arn* operon expression without affecting *pmrB* expression. In contrast, PmrB mutations increased both *arn* and *speD2* operon expression (black arrows) (see Fig. 4). Adaptation to AMPs, according to RNA-seq experiments, likely occurs through induction of *arn* and *speD2* operons via ParRS (Pmx-B) or CprRS (dendrimers) TCSs (see Table 5). Induction of these operons may occur either directly (dashed gray lines) or through activation of *pmrAB* expression (not shown).

To further investigate the response of P. aeruginosa to Pmx-B and G3KL, we performed a transcriptome analysis by RNA-seq of the PA14 wild type exposed to subinhibitory concentrations (0.25× the MIC) of Pmx-B, G3KL, or T7. Overall, Pmx-B exposure resulted in a broader response compared to dendrimers. Indeed, PA14 responded to Pmx-B by upregulating 73 and downregulating 108 genes. In comparison, G3KL and T7 induced, respectively, upregulation of 29 and 21 genes and downregulation of 18 and 27 genes (Tables S4, S5 and S6). Not unexpectedly, genes that were upregulated by all three AMPs included the complete *arn* operon genes as well as those of the *speD2* operon (Table 5). Interestingly, T7 showed the highest induction of the *arn* operon (8- to 12-fold change), while Pmx-B and G3KL elicited a lower response (3- to 4-fold change). Expression of the *speD2* operon was induced 4- to 15-fold by Pmx-B and T7 and only 3-fold by G3KL. As expected from our quantitative real-time PCR (qPCR) data (Fig. 4b), expression of the *pmrAB* genes, which regulate both *arn* and *speD2* operons, were only marginally upregulated by the AMPs. Interestingly, two other loci showed marked changes in gene expression, namely, PA14_24360 (no homologue in PAO1), a gene located next to the *cprRS* operon, and PA14_41280 (PA1797), located next to the *parRS* operon. While the former was induced only by the dendrimers, the latter responded strongly (54-fold upregulation) to Pmx-B but not to the dendrimers (<2-fold). We also observed an 8- to 9-fold induction by Pmx-B of the *mexXY* genes, encoding the MexXY efflux pump involved in aminoglycoside export (31). Two other genes were induced by the three AMPs: (i) the *cprA* gene, encoding a protein of unknown function containing a short-chain dehydrogenase/isomerase domain (SDR) and shown to be involved in Pmx-B resistance in P. aeruginosa strain PAK (32) and (ii) the *carO* gene, encoding a periplasmic protein required for calcium homeostasis (33). In summary, our transcriptomic data suggest that the same LPS-modification mechanisms are expressed in response to the three AMPs as those overexpressed in the *pmrB* mutants. However, Pmx-B seems to signal via the ParRS TCS, while the dendrimers specifically induce the CprRS TCS (Fig. 5).

**TABLE 5:** Gene expression profiling in response to AMPs in PA14

PA 14 locus	Gene/locus^*a*^	Expression fold change^*b*^		
		Pmx-B	G3KL	T7
PA14_18350	***arnA***	3.1	3.7	10.0
PA14_18370	***arnB***	2.1	3.4	8.1
PA14_18360	***arnC***	3.2	4.4	10.4
PA14_18340	***arnD***	3.3	3.7	11.1
PA14_18320	***arnE***	3.1	3.6	9.5
PA14_18310	***arnF***	3.4	3.2	10.1
PA14_18330	***arnT***	3.3	3.6	10.4
PA14_18300	***ugd***	3.8	3.9	11.5
PA14_04180	*carO*	4.6	2.6	5.5
PA14_44311	*cprA*	3.8	5.3	15.3
PA14_63110	***speD2***	13.7	2.9	8.1
PA14_63120	***speE2***	15.2	3.6	9.7
PA14_63130	**PA4775**	5.1	NC	3.9
PA14_24360	next *cprRS*	2.0	9.2	28.8
PA14_41280	next *parRS*	54.0	NC	2.2
PA14_38395	*mexX*	8.3	NC	NC
PA14_38410	*mexY*	8.9	NC	NC
PA14_63150	*pmrA*	2.6	NC	2.4
PA14_63160	*pmrB*	2.3	NC	2.2

a Genes in bold belong to the *arn* and *spe* operons.

b NC, no change.

## DISCUSSION

The purpose of this study was to unravel potential resistance mechanisms against dendrimer AMPs, which differ in size, number of charges, and structure from the cyclic polymyxins and linear antimicrobial peptides of mammalian origin. A major difference was the difficulty in obtaining spontaneously resistant mutants with both dendrimers, where we estimate the selection frequency with G3KL was at least 10- to 100-fold lower than with Pmx-B. This might be due to faster killing of P. aeruginosa by G3KL compared to Pmx-B, or to the requirement of specific mutations located in the periplasmic domain of the PmrB sensor kinase gene. The fact that 20% of spontaneous mutants selected on Pmx-B also showed decreased susceptibility to G3KL, but not to T7, indicates that only partial cross-resistance exists between Pmx-B and certain dendrimers. Indeed, analysis of additional dendrimer peptides revealed heterogeneity in their MICs for the selected mutants (data not shown). All of the mutants resistant only to Pmx-B carried exclusively loss-of-function mutations in the sensor kinase PhoQ of the PhoPQ two-component system, as observed in polymyxin-resistant clinical isolates of P. aeruginosa (34–36). These mutants are characterized by upregulation of the *arnBCADTEF-ugd* operon, leading to addition of lipid A by l-4AraN (27). This modification alone, however, had no effect on dendrimer susceptibilities. Only mutations that occurred more rarely in the PmrB TCS sensor kinase also affected G3KL activities. Intriguingly, all mutations affecting dendrimer MICs carried specific amino acid substitutions in the periplasmic loop of PmrB, sensing the periplasmic concentration of divalent cations (Mg^2+^ and Ca^2+^) (26). These mutations likely result in cation-independent activation of the PmrAB signaling cascade leading to increased expression of one of its specific targets, namely the operon *speD2-speE2*-PA4775, located upstream of *pmrAB*. Indeed, Mg^2+^ ion starvation was shown to strongly induce expression of the *speD2* operon (28). The s*peD2* gene encodes an S-adenosylmethionine decarboxylase and *speE2*, a putative spermidine synthase. The protein encoded by PA4775 has no homologue within the databases but the open reading frame (ORF) is cotranscribed with *speE2* (28) and is required for conferring resistance to dendrimers. Indeed, the *speD2* operon was recently described as being responsible for the synthesis of norspermidine (29, 37, 38). However, addition of spermidine or norspermidine, carrying three amine groups, had no effect on AMP activities. In contrast, spermine and norspermine, both harboring four amine groups, increased Pmx-B and dendrimer MICs but only in a mutant overexpressing the *arn* operon. Lipid A modification by l-Ara4N therefore seems to be a prerequisite for polyamines to exert their protective effect. The exact site and type of interaction between the polyamines and lipid A remains to be determined. l-Ara4N addition occurs either on one or on both moieties of the D-glucosamine disaccharide of lipid A, leaving two to three additional phosphate groups on the 2-keto-3-deoxyoctulosonic acid (KDO) core sugars available for potential interactions with polyamines (39). The secreted polyamines could stabilize the outer membrane under low Mg^2+^ ion conditions and thereby interfere with binding of positively charged antibiotics, including aminoglycosides (29), polymyxins, and the dendrimers. The dendrimers are predicted to form a globular structure with hydrophilic residues exposed to the exterior and the hydrophobic residues buried inside the molecule (21). Upon contact with a hydrophobic environment, the structure would unfold to expose the hydrophobic residues for interaction with the phospholipids of the outer membrane. We expect that the polyamines reduce initial binding of G3KL to the phosphates of the core LPS sugars, thereby decreasing subsequent insertion into the lipid layer, unfolding, and translocation into the periplasm.

Transcriptomic analysis and time-kill assays show that G3KL and T7 induce an adaptive response in PA14 by increasing expression of the same gene products (*arn* and *speD2* operons) as those overexpressed in the *pmrB* mutants. However, the response to G3KL was different between the three *pmrB* mutants. While the mutant strains 4.18 (PmrB Thr132Pro) and 4.19 (PmrB Phe124Cys) still responded to G3KL, as indicated by induction of the *arnB* gene, the PmrB D47E mutation present in mutant 2P4 resulted in constitutive high-level expression of *arnB* independent of G3KL (Fig. 4a). The transcriptome analysis further suggests that the dendrimers trigger the CprRS TCS while Pmx-B signals through the ParRS TCS, as described previously (31, 40) (Fig. 5). CprRS is a peptide-responsive TCS, however, only linear peptides were tested and included molecules with 3 to 7 positive charges and 7 to 34 amino acids (41). It is therefore surprising that CprS also recognizes dendrimers, presenting 15 to 17 positive charges and a branched-chain structure with 37 to 40 amino acids. This might be due to the higher number of negative charges present in the periplasmic domain of CprS (28 Asp/Glu residues, including a consecutive stretch of ten Asp/Glu), compared to PmrB (19 Asp/Glu) and PhoQ (22 Asp/Glu), which are likely specialized in sensing divalent cations (26).

In summary, peptide dendrimers are promising antimicrobial compounds with variable structures and a low propensity for selection of resistance. Although P. aeruginosa shows similar mechanisms involved in protection against dendrimers as for polymyxins, the rapid killing observed with G3KL and the requirement for very specific *pmrB* mutations make them an interesting option for future development as novel therapeutic agents against MDR Gram-negative bacteria.

## MATERIALS AND METHODS

### Bacterial strains, media, and chemicals.

Bacterial strains and plasmids used in this study are shown in Table 6. The primers used for qPCR analyses and construction of gene knockouts are listed in Table S3. P. aeruginosa and Escherichia coli strains were grown in lysogeny broth (LB) at 37°C with agitation (250 rpm). E. coli strain DH10B was used as a host for cloning experiments.

**TABLE 6:** Bacterial strains and plasmids used in this study

Strain or plasmid	Relevant characteristics	Source or reference
*P. aeruginosa* strains		
PA14	wild-type	(46)
4.13	PA14 phoQ 1105ins4bp, V369fs (Pmx-B selected)	This study
4.18	PA14 pmrB 394A>C, T132P (Pmx-B selected)	This study
4.19	PA14 pmrB 371T>G, F124C (Pmx-B selected)	This study
2P4	PA14 pmrB A141G, D47E (T7 selected)	This study
PA14 ΔarnT	Unmarked deletion of *arnT* gene	This study
4.13 ΔarnT	Unmarked deletion of *arnT* gene	This study
4.18 ΔarnT	Unmarked deletion of *arnT* gene	This study
4.18 ΔPAspeE2	Unmarked deletion of *speE2* (PA4774) locus	This study
4.19 ΔPAspeE2	Unmarked deletion of *speE2* (PA4774) locus	This study
*E. coli* strains		
S17-1λ*pir*	Strain used for conjugation	(47)
DH10B	F– *endA*1 *deoR*+ *recA*1 *galE*15 *galK*16 *nupG rpsL* Δ(*lac*)X74 φ80*lacZ*ΔM15 *araD*139 Δ(ara,leu)7697 *mcrA* Δ(*mrr-hsdRMS-mcrBC*), Sm^R^, λ–	Lab collection
Plasmids		
pEXG2	Suicide vector, Gm	(48)
pIApX2	pUCP20 derivative, cloning vector with constitutive promoter; *gfp*; Ap	I. Attree, France
pspD2	pIApX2 carrying *speD2* gene from PA14	This study
pspE2	pIApX2 carrying *speE2* gene from PA14	This study
pPA4775	pIApX2 carrying *PA4775* gene from PA14	This study
pspDE2	pIApX2 carrying *speD2* and *speE2* genes from PA14	This study
pspE2-5	pIApX2 carrying *speE2* and PA4775 genes from PA14	This study
pspDE2-5	pIApX2 carrying the *speD2-speE2*-PA4775 operon from PA14	This study
pΔarnT	pExG2 derivative used to create *arnT* deletion mutant	This study
pΔspE2	pExG2 derivative used to create *speE2* deletion mutant	This study

Dendrimers were synthesized and purified as described (19, 42). Polymyxin-B, polyamines, and chemicals were purchased from Merck-Sigma (Switzerland).

### Selection of mutants with decreased susceptibility to Pmx-B and dendrimers.

Spontaneous P. aeruginosa mutants resistant to polymyxin B (Pmx-B) were selected on Mueller-Hinton broth (MHB) agar plates supplemented with 4 mg/liter of Pmx-B. An 150-μL aliquot of P. aeruginosa culture grown in LB medium was spread on these plates and incubated overnight at 37°C and then at room temperature until the appearance of P. aeruginosa colonies.

### Whole-genome sequencing.

Genomic DNA was extracted using the DNeasy blood and tissue kit (Qiagen) from bacterial strains grown in LB medium to midexponential phase (approximately to optical density at 600 nm (OD_600_) of 1.0). DNA libraries were sequenced on an Illumina HiSeq instrument (100-bp paired-end reads) at the iGE3 Genomics platform of the University of Geneva.

### MIC determinations.

MICs for all strains were determined in Mueller-Hinton broth (MHB) by broth microdilution in accordance to the Clinical and Laboratory Standards Institute (CLSI) guidelines (43). AMP concentrations ranged from 0.125 to 16 mg/liter (Pmx-B) and 0.5 to 64 mg/liter (G3KL and T7). MICs were performed at least three times. Polyamines were dissolved in distilled water (dH_2_O) and added at the indicated concentrations.

### Killing kinetic assay.

Bacteria were grown overnight in LB medium. For the Pmx-B and G3KL preincubation, bacteria were grown with 0.25× MIC of the corresponding compound. Next, 500 μl of culture was centrifuged at 5,000 rpm for 3 min and pellets were resuspended in M9-salts medium and adjusted to OD_600_ = 2.0. Fifteen microliters from these bacterial suspensions were added to the first row of a microtiter plate containing 135 μl of M9-salts medium (Na_2_HPO_4_ 6 g/liter; KH_2_PO_4_ 3 g/liter; NaCl 0.5 g/liter; NH_4_Cl 1 g/liter) supplemented with 2 mM MgSO_4_ and either 6 mg/liter of Pmx-B or 48 mg/liter of G3KL. The microtiter plates were incubated at 37°C. At each time point (10 min, 30 min, 1 h, 4 h, and 24 h), serial dilutions were spotted in triplicate onto LB agar plates and CFU counts were determined after incubation at 37°C.

### RNA extraction.

For RNA extraction, the strains were grown under MIC assay conditions in microtiter plates in MH broth, supplemented or not with 0.25× the MIC of Pmx-B, G3KL, or T7. Three wells were pooled to form one sample. RNA was extracted using the RNeasy minikit (Qiagen) according to the manufacturer’s instructions. Genomic DNA was removed by treatment with RNase-free DNase (Promega). Five hundred nanograms of RNA was reverse transcribed using ImProm-II reverse transcriptase (Promega).

### Quantitative real-time PCR.

Gene expression was measured using a Rotor-Gene SYBR Green PCR kit (Qiagen), with primers specific to the genes of interest and the *rpsL* housekeeping gene (44). qPCRs were performed in a Rotor-Gene 3000 (Corbett Research) with the following conditions: 5 min at 95°C, 5 sec at 95°C, and 10 sec at 60°C (40 cycles). A melt-curve analysis was performed (60°C to 99°C) to confirm the presence of a single amplification product.

### RNA sequencing and bioinformatics analysis.

Approximately 1.3 μg of total RNA for each replicate sample was ribodepleted using the Ribo-Zero rRNA removal kit for bacteria (Epicentre) according to the manufacturer’s protocol. The TruSeq total RNA stranded kit (Illumina) was used for the preparation of libraries. A Quibit spectrophotometer measured their quantity and a Tapestation on a DNA High-sensitivity chip (Agilent Technologies) determined their quality. The 12 generated libraries were pooled at equimolarity and loaded at 7 pM for clustering. An Illumina HiSeq 4000 instrument was used to sequence 100-bp single reads per sample (iGE3 Genomics platform of the University of Geneva). FastQC v.0.11.5 was used for sequencing quality control and reads were mapped to the NCBI NC_008463.1 Pseudomonas aeruginosa UCBP-PA14 reference genome using BWA aligner v.0.7.17 software. The counts were normalized according to the relative number of reads for each library, and genes having a count above 1 count per million reads (cpm) in at least 3 samples were kept for the analysis. The starting raw gene number of the set was 6,100. The final filtered data set consisted of 6,043 genes after excluding poorly or nonexpressed genes.

### Construction of deletion mutants.

The upstream and downstream flanking regions of the target locus were PCR amplified (usually 500 to 600 bp) using primers with HindIII/BamHI or BamHI/EcoRI restriction sites for the upstream and downstream flanking regions, respectively. DNA fragments were ligated and inserted into the suicide vector pEXG2 (45) using HindIII and EcoRI restriction sites. Plasmid constructs were transferred into the E. coli mobilizing strain S17-λpir. After conjugation into P. aeruginosa, mero-diploids were selected on M9-salts medium plates supplemented with citrate (20 mM) and gentamicin (50 mg/liter). To select for recombinants which had lost the integrated plasmid, cell suspensions were streaked on M9-salts agar plates supplemented with 20 mM citrate and 10% sucrose. Sucrose-resistant and gentamicin-susceptible colonies were screened by PCR for the presence of the expected deletion in the corresponding target gene. Two independent clones for each mutant were selected for further analyses and frozen as glycerol stocks at −80 °C.

### Construction of expression plasmids.

The DNA regions with at least 60 nucleotides upstream of the ATG initiation codon and downstream of the STOP codon were amplified by PCR from genomic DNA of P. aeruginosa strain PA14. All primers contained BamHI and HindIII restriction sites. DNA fragments were cloned into the expression vector pIApX2 (Table 6). Plasmid constructs were verified by Sanger sequencing. Plasmids were transferred into P. aeruginosa by electroporation and transformants were selected on LB agar plates supplemented with 200 mg/liter of carbenicillin. Two independent colonies were chosen for further analyses.

### Availability of data and materials.

The meta-data and raw data generated in this study are available at GEO under the following series record: GSE139257.

## Supplementary Material

Supplemental file 1
