# Supplementary material for: Adaptive and Mutational Responses to Peptide Dendrimer Antimicrobials in Pseudomonas aeruginosa
Source: Antimicrob Agents Chemother. 2020 Mar 24;64(4):e02040-19. doi: 10.1128/AAC.02040-19 (PMC7179292; doi:10.1128/AAC.02040-19)
Supplement: Supplemental file 1 [file AAC.02040-19-s0001.pdf]

Table S1. PA14-derived mutants selected on Pmx-B (4 mg/L)

Strain	Selection	MIC (mg/L)		gene	nt, aa change
		Pmx-B	G3KL		
PA14	none	1	8	*phoQ*	wt
				pmrB	wt
4.1	Pmx-B	8	8	*phoQ*	735_738del, Asp244fs
				pmrB	wt
4.2	Pmx-B	8	8	*phoQ*	1142A>G, Trp381X
				pmrB	wt
4.3	Pmx-B	8	8	*phoQ*	5insT, Ile2fs
				pmrB	wt
4.4	Pmx-B	8	8	*phoQ*	41_165del, Ala14fs
				pmrB	ND
4.5	Pmx-B	8	8	*phoQ*	41_165del, Ala14fs
				pmrB	ND
4.6	Pmx-B	8	8	*phoQ*	41_165del, Ala14fs
				pmrB	ND
4.8	Pmx-B	8	8	*phoQ*	41_165del, Ala14fs
				pmrB	wt
4.9	Pmx-B	8	8	*phoQ*	41_165del, Ala14fs
				pmrB	wt
4.10	Pmx-B	8	8	*phoQ*	41_165del, Ala14fs
				pmrB	wt
4.11	Pmx-B	8	8	*phoQ*	41_165del, Ala14fs
				pmrB	wt
**4.13**	Pmx-B	8	8	***phoQ***	1105insCGCG,Val369fs
				pmrB	wt
4.14	Pmx-B	8	8	*phoQ*	41_165del, Ala14fs
				pmrB	wt
4.15	Pmx-B	8	8	*phoQ*	41_165del, Ala14fs
				pmrB	wt
4.16	Pmx-B	8	8	*phoQ*	41_165del, Ala14fs
				pmrB	ND
4.17	Pmx-B	8	8	*phoQ*	41_165del, Ala14fs
				pmrB	wt

**4.18**	Pmx-B	8	32	*phoQ*	wt
				pmrB	394A>C, Thr132Pro
**4.19**	Pmx-B	16	32	*phoQ*	wt
				pmrB	371T>G, Phe124Cys

nt, nucleotide; aa, amino acid; ND, not determined
strains selected for this study are highlighted in bold

Table S2. Effect of *speD2* operon gene expression on AMP activity

	MIC (mg/L)	
Strain	Pmx-B	G3KL
PA14	1	8
PA14 vector	1	8
PA14 pspD2	1	8
PA14 pspE2	1	8
PA14 pPA4775	1	8
PA14 pspDE2	1	8
PA14 pspE2-5	1	8
PA14 pspDE2-5	1	16
4.13 (*phoQ*)	8	8
4.13 vector	8	8
4.13 pspD2	8	8
4.13 pspE2	8	8
4.13 pPA4775	8	8
4.13 pspDE2	8	8
4.13 pspE2-5	8	8
4.13 pspDE2-5	8	**>64**

Table S3. Primers used in this study

Primer	Sequence (5'-3')	Source
arnB3	GCCGAAGAACCAGGAGCTTG	This study
arnB4	CGGTGGAGACCCAGGTCAG	This study
pmrB1	CCTCTCGCTGAAGCAGGTGA	This study
pmrB2	CTGGTCTTCGGTGGCAAGGT	This study
PA4773-1	CTCGGCAAGCAACTGGTCAT	This study
PA4773-2	AACTTGTGGAACGCCGAGGT	This study
PA4774-1	GAGTTCTCCCACAGCGACGA	This study
PA4774-2	AGCCCCAGGAGGAGAGGAAC	This study
arnT-HindIII-F1	ACACAAGCTTCCTTCGGCTTCCGCTACAAC	This study
arnT-BamHI-R1	ACACGGATCCGCGACCGGCTTCTCGAAGTA	This study
arnT-BamHI-F2	ACACGGATCCCAGAACAAGACCCCCGACCT	This study
arnT-EcoRI-R2	ACACGAATTCACTTCCACCCGTTGCAGGA	This study
PA4774-HindIII-F1	ACACAAGCTTGCAATTCAACCGTTGCGACT	This study
PA4774-BamHI-R1	ACACGGATCCGGTCCGGCGGATAGAAATAGA	This study
PA4774-BamHI-F2	ACACGGATCCTACCTCAAGGCCTGCTTCGT	This study
PA4774-EcoRI-R2	ACACGAATTCGGTAGCTGGGTCAGTTCGACA	This study
PA4773-BamHI-F	ACACGGATCCGTGTCCACCAGCCGTACCTG	This study
PA4773-HindIII-R	ACACAAGCTTCGCAGGCGGTAGACGTACTG	This study
PA4774-BamHI-F	ACACGGATCCTGATGGATACGCCGATCGAA	This study
PA4774-HindIII-R	ACACAAGCTTAGTCGGAAGCCAGCAGGAAG	This study
PA4775-BamHI-F	ACACGGATCCGGGACCGGTACTGGAAGACG	This study
PA4775-HindIII-R	ACACAAGCTTGATCGAAGGGCTTGGTCAGG	This study

Table S4 RNAseq transcriptome analysis of PA14 in response to Pmx-B exposure (0.25 x MIC)

PA14 locus	logFC	FC	gene	description
PA14_09240	-5.37	0.02	pchD	2,3-dihydroxybenzoate-AMP ligase (EC 2.7.7.58) [pyochelin] siderophore
PA14_09230	-5.33	0.02	pchC	Pyochelin biosynthetic protein PchC, predicted thioesterase
PA14_09220	-5.15	0.03	pchB	Isochorismate pyruvate-lyase (EC 4.2.99.21) [pyochelin] siderophore
PA14_09270	-5.06	0.03	pchE	Dihydroaeruginoate synthetase PchE, non-ribosomal peptide synthetase modules
PA14_09340	-5.01	0.03	fptA	Outer membrane receptor for ferric-pyochelin FptA
PA14_09290	-5.00	0.03	pchG	Pyochelin biosynthetic protein PchG, oxidoreductase (NAD-binding)
PA14_09280	-4.99	0.03	pchF	Pyochelin synthetase PchF, non-ribosomal peptide synthetase module
PA14_09210	-4.97	0.03	pchA	Isochorismate synthase (EC 5.4.4.2)
PA14_09380	-4.85	0.03	ampP, fptX	Inner-membrane permease FptX, ferripyochelin
PA14_09320	-4.84	0.03	pchI	ABC efflux pump, fused inner membrane and ATPase subunits in pyochelin gene cluster
PA14_09300	-4.78	0.04	pchH	ABC efflux pump, fused inner membrane and ATPase subunits in pyochelin gene cluster
PA14_09370	-4.76	0.04	ampO	
PA14_09350	-4.44	0.05	fptB	Hypothetical protein FtpB in pyochelin gene cluster
PA14_19590	-3.26	0.10	ssuF	Organosulfonate utilization protein SsuF
PA14_19580	-2.47	0.18	ssuB	Alkanesulfonate ABC transporter ATP-binding protein SsuB
PA14_19560	-2.46	0.18	ssuD	FMNH2-dependent alkanesulfonate monooxygenase (EC 1.14.14.5)
PA14_34770	-2.39	0.19	tauA	Taurine ABC transporter, substrate-binding protein TauA
PA14_22320	-2.28	0.21		Uncharacterized membrane protein SO_4740
PA14_19570	-2.16	0.22	ssuC	Alkanesulfonate ABC transporter permease protein SsuC
PA14_34780	-2.14	0.23	tauB	Taurine ABC transporter, ATP-binding protein TauB
PA14_02500	-2.13	0.23	exbB1	MotA/TolQ/ExbB proton channel family protein
PA14_58040	-2.04	0.24		FagA protein
PA14_58030	-2.00	0.25	fumC	Fumarate hydratase class II (EC 4.2.1.2)
PA14_58010	-1.98	0.25		hypothetical protein
PA14_60480	-1.96	0.26		hypothetical protein
PA14_12970	-1.94	0.26	tauD	Alpha-ketoglutarate-dependent taurine dioxygenase (EC 1.14.11.17)
PA14_33270	-1.92	0.26	pvdG	Thioesterase PvdG involved in non-ribosomal peptide biosynthesis

PA14_58000	-1.92	0.26	sodM	Superoxide dismutase [Mn] (EC 1.15.1.1)
PA14_33510	-1.88	0.27	fpvF	MbtH-like NRPS chaperone
PA14_02490	-1.87	0.27	tonB2	putative TonB-dependent receptor
PA14_33730	-1.75	0.30	pvdM	Putative dipeptidase, pyoverdin biosynthesis PvdM
PA14_20020	-1.72	0.30	hasAp	Hemophore HasA
PA14_33280	-1.71	0.31	pvdL	Pyoverdine chromophore precursor synthetase PvdL
PA14_02510	-1.68	0.31	exbD1	Biopolymer transport protein ExbD/TolR
PA14_61200	-1.68	0.31		Large exoproteins involved in heme utilization or adhesion
PA14_21530	-1.67	0.31		FOG: Ankyrin repeat
PA14_33520	-1.64	0.32	fpvE	FIG057993:Thioesterase involved in non-ribosomal peptide biosynthesis
PA14_12940	-1.61	0.33	tauB	Taurine ABC transporter, ATP-binding protein TauB
PA14_33710	-1.59	0.33	pvdO	PvdO, pyoverdine responsive serine/threonine kinase (predicted by OlgaV)
PA14_35240	-1.55	0.34		Uncharacterized protein Pfl_4311 / Uncharacterized protein YqjZ
PA14_19540	-1.55	0.34	ssuA	Alkanesulfonate ABC transporter substrate-binding protein SsuA
PA14_39420	-1.54	0.34		hypothetical protein
PA14_33820	-1.51	0.35	pvdQ	Acyl-homoserine lactone acylase PvdQ (EC 3.5.1.-), quorum-quenching
PA14_12960	-1.50	0.35	tauC	Taurine ABC transporter, permease protein TauC
PA14_01720	-1.50	0.35	ahpF	Alkyl hydroperoxide reductase protein F
PA14_09540	-1.48	0.36	mexG	hypothetical protein
PA14_57990	-1.47	0.36		Metal transporter, ZIP family
PA14_02420	-1.46	0.36		Aliphatic sulfate esters (C4-C12 chain lengths) dioxygenase (EC 1.14.11.-)
PA14_34750	-1.45	0.37		Alpha-ketoglutarate-dependent taurine dioxygenase (EC 1.14.11.17)
PA14_34790	-1.42	0.37	tauC	Taurine ABC transporter, permease protein TauC
PA14_24860	-1.39	0.38	snr1	Cytochrome c
PA14_61190	-1.37	0.39		Hemolysin activation/secretion protein
PA14_35160	-1.36	0.39		hypothetical protein
PA14_47400	-1.36	0.39		FIG006045: Sigma factor, ECF subfamily
PA14_33810	-1.35	0.39	pvdA	L-ornithine 5-monooxygenase (EC 1.13.12.-), PvdA of pyoverdin biosynthesis
PA14_37980	-1.35	0.39		Putative transmembrane sensor
PA14_01710	-1.34	0.39	ahpC	Alkyl hydroperoxide reductase protein C (EC 1.11.1.15)

PA14_64700	-1.34	0.40		FIG006045: Sigma factor, ECF subfamily
PA14_55580	-1.34	0.40	nemO	Heme oxygenase HemO, associated with heme uptake
PA14_09530	-1.32	0.40	mexH	Multidrug efflux system, membrane fusion component
PA14_33260	-1.31	0.40	pvdS	Sigma factor PvdS, controling pyoverdin biosynthesis
PA14_33540	-1.29	0.41	fpvD	ABC transporter in pyoverdin gene cluster, permease component
PA14_61040	-1.28	0.41	katB	Catalase KatE (EC 1.11.1.6)
PA14_33690	-1.27	0.41	pvdE	PvdE, pyoverdine ABC export system, fused ATPase and permease components
PA14_45100	-1.26	0.42		hypothetical protein
PA14_47390	-1.26	0.42		Iron siderophore sensor protein
PA14_33500	-1.25	0.42	pvdH	Pyoverdin biosynthesis protein PvdH, L-2,4-diaminobutyrate:2-oxoglutarate aminotransferase
PA14_09260	-1.24	0.42	pchR	Transcriptional regulator PchR
PA14_13010	-1.24	0.42		Methionine ABC transporter substrate-binding protein
PA14_37990	-1.24	0.42		RNA polymerase ECF-type sigma factor
PA14_38220	-1.22	0.43		Siderophore-interacting protein
PA14_33560	-1.21	0.43	fpvC	FIG014801: Cation ABC transporter, periplasmic cation-binding protein
PA14_12920	-1.21	0.43	tauA	Taurine ABC transporter, substrate-binding protein TauA
PA14_40260	-1.21	0.43		T1SS secreted agglutinin RTX
PA14_28970	-1.16	0.45		hypothetical protein
PA14_10370	-1.15	0.45		FAD/FMN-containing dehydrogenases
PA14_64530	-1.15	0.45		hypothetical protein
PA14_40230	-1.14	0.45		Type I secretion system, membrane fusion protein LapC
PA14_34740	-1.13	0.46		hypothetical protein
PA14_33580	-1.11	0.46	fpvJ	FIG137877: Hypothetical protein in pyoverdin gene cluster
PA14_13000	-1.11	0.46		sigma-54-dependent transcriptional regulator
PA14_09520	-1.10	0.47	mexI	Multidrug efflux system, inner membrane proton/drug antiporter (RND type)
PA14_33680	-1.10	0.47	fpvA	Outer membrane ferripyoverdine receptor FpvA, TonB-dependent
PA14_53300	-1.10	0.47		Alkyl hydroperoxide reductase subunit C-like protein
PA14_71100	-1.10	0.47		UPF0324 inner membrane protein YeiH
PA14_34730	-1.10	0.47		Transcriptional regulator, Xre-family with cupin domain
PA14_33570	-1.09	0.47	fpvK	FIG049111: Hypothetical protein in pyoverdin gene cluster

PA14_10170	-1.09	0.47	fepB	Ferrienterobactin-binding periplasmic protein FepB (TC 3.A.1.14.2)
PA14_46030	-1.08	0.47		Methyl-accepting chemotaxis sensor/transducer protein
PA14_22980	-1.08	0.47		Glucose ABC transport system, periplasmic sugar-binding protein
PA14_39780	-1.07	0.47		NAD glycohydrolase, hvnA
PA14_64690	-1.07	0.48		Iron siderophore sensor protein
PA14_33610	-1.07	0.48	pvdI	Pyoverdine sidechain non-ribosomal peptide synthetase PvdI
PA14_40240	-1.07	0.48		Type I secretion system ATPase, LssB family LapB
PA14_33600	-1.06	0.48	fpvG	FIG137594: Putative iron-regulated membrane protein
PA14_10380	-1.06	0.48		hypothetical protein
PA14_03700	-1.05	0.48	sbp	Sulfate and thiosulfate binding protein CysP
PA14_09500	-1.05	0.48	opmD	Multidrug efflux system, outer membrane factor lipoprotein
PA14_34020	-1.04	0.49	tssE	T6SS lysozyme-like component TssE
PA14_03680	-1.04	0.49	cysT	Sulfate transport system permease protein CysT
PA14_03710	-1.03	0.49		Uncharacterized protein PA0284
PA14_47920	-1.02	0.49		4-hydroxyproline ABC transporter, substrate-binding protein
PA14_37470	-1.01	0.50		Coenzyme F420-dependent N5,N10-methylene tetrahydromethanopterin reductase
PA14_30550	-1.01	0.50		ABC transporter, substrate-binding protein (cluster 10, nitrate/sulfonate/bicarbonate)
PA14_26020	-1.00	0.50		Uncharacterized lipoprotein aminopeptidase LpqL
PA14_58330	-1.00	0.50		probable periplasmic protein NMA1059
PA14_33250	-1.00	0.50	pvdY	Hypothetical protein PvdY
PA14_24360	1.00	2.00		next CprRS, hypothetical protein
PA14_06730	1.01	2.01	nirC	Cytochrome c55X precursor NirC
PA14_06680	1.01	2.01	nirH	Heme d1 biosynthesis protein NirH
PA14_70690	1.01	2.02	glcD	Glycolate dehydrogenase (EC 1.1.99.14), subunit GlcD
PA14_58570	1.02	2.03		Ferrichrome-iron receptor
PA14_54540	1.02	2.03		Tripartite tricarboxylate transporter TctC family
PA14_06670	1.04	2.05	nirJ	Heme d1 biosynthesis protein NirJ
PA14_06830	1.04	2.06	norB	Nitric-oxide reductase subunit B (EC 1.7.99.7)
PA14_06720	1.06	2.09	nirF	Heme d1 biosynthesis protein NirF
PA14_06740	1.06	2.09	nirM	Cytochrome c551 NirM

PA14_06650	1.08	2.11	nirN	Nitrite reductase associated c-type cytochorome NirN
PA14_06660	1.08	2.12	nirE	Uroporphyrinogen-III methyltransferase (EC 2.1.1.107)
PA14_18370	1.09	2.12	arnB	UDP-4-amino-4-deoxy-L-arabinose--oxoglutarate aminotransferase (EC 2.6.1.87)
PA14_44240	1.10	2.14		glutamine synthetase family protein
PA14_06810	1.10	2.14	norC	Nitric-oxide reductase subunit C (EC 1.7.99.7)
PA14_67150	1.13	2.19		D-amino acid dehydrogenase (EC 1.4.99.6)
PA14_72180	1.14	2.20		UPF0053 inner membrane protein YgdQ
PA14_11140	1.16	2.23		Polyketide synthase modules and related proteins
PA14_54550	1.17	2.25		Tripartite tricarboxylate transporter TctB family
PA14_44260	1.18	2.26		O-acetylhomoserine sulfhydrylase (EC 2.5.1.49)
PA14_63220	1.19	2.28		hypothetical protein
PA14_54570	1.21	2.31		Tripartite tricarboxylate transporter TctA family
PA14_18260	1.22	2.34	fruK	1-phosphofructokinase (EC 2.7.1.56)
PA14_63160	1.23	2.35	pmrB	Sensory histidine kinase QseC
PA14_06710	1.24	2.36	nirD	Heme d1 biosynthesis protein NirD
PA14_54580	1.25	2.37		Ammonia monooxygenase
PA14_18250	1.26	2.40	fruI	PTS system, fructose-specific IIA component (EC 2.7.1.202) / Phosphotransferase
PA14_18275	1.29	2.45	fruA	PTS system, fructose-specific IIB component (EC 2.7.1.202) / PTS system
PA14_12990	1.32	2.49	betT	High-affinity choline uptake protein BetT
PA14_06840	1.36	2.56	norD	Nitric oxide reductase activation protein NorD
PA14_70650	1.38	2.60	glcG	Hypothetical protein GlcG in glycolate utilization operon
PA14_70670	1.38	2.60	glcF	Glycolate dehydrogenase (EC 1.1.99.14), iron-sulfur subunit GlcF
PA14_63150	1.40	2.64	pmrA	Two-component system response regulator QseB
PA14_RS04385	1.47	2.77		NAD(P)-dependent oxidoreductase
PA14_52840	1.50	2.83		TRAP-type C4-dicarboxylate transport system, periplasmic component
PA14_70980	1.56	2.95	betT1	High-affinity choline uptake protein BetT
PA14_18320	1.63	3.09	arnE	Undecaprenyl phosphate-aminoarabinose flippase subunit ArnE
PA14_18350	1.65	3.13	arnA	UDP-4-amino-4-deoxy-L-arabinose formyltransferase (EC 2.1.2.13)
PA14_18360	1.70	3.24	arnC	Undecaprenyl-phosphate 4-deoxy-4-formamido-L-arabinose transferase (EC 2.4.2.53)
PA14_18340	1.71	3.26	arnD	4-deoxy-4-formamido-L-arabinose-phosphoundecaprenol deformylase ArnD (EC 3.5.1.n3)

PA14_52870	1.73	3.31		acyl CoA transferase ; CaiB/BaiF family protein
PA14_18330	1.74	3.33	arnT	Undecaprenyl phosphate-alpha-4-amino-4-deoxy-L-arabinose arabinosyl transferase
PA14_18310	1.75	3.37	arnF	Undecaprenyl phosphate-aminoarabinose flippase subunit ArnF
PA14_52850	1.83	3.56		citrate lyase subunit, beta chain, 3
PA14_20170	1.87	3.65	nosY	Nitrous oxide reductase maturation transmembrane protein NosY
PA14_44311	1.90	3.75	cprA	Nucleoside-diphosphate-sugar epimerases
PA14_18300	1.91	3.75	ugd	UDP-glucose 6-dehydrogenase (EC 1.1.1.22)
PA14_34210	1.91	3.76		T6SS sigma-54-dependent regulator VasH
PA14_20200	1.97	3.93	nosZ	Nitrous-oxide reductase (EC 1.7.99.6) / Pseudoazurin
PA14_70950	2.01	4.02	betB	Betaine aldehyde dehydrogenase (EC 1.2.1.8)
PA14_20190	2.05	4.15	nosD	Nitrous oxide reductase maturation protein NosD
PA14_34200	2.07	4.21	msuC	Acyl-CoA dehydrogenase; probable FMNH2-dependent monooxygenase
PA14_20180	2.08	4.22	nosF	Nitrous oxide reductase maturation protein NosF (ATPase)
PA14_70940	2.08	4.22	betA	Choline dehydrogenase (EC 1.1.99.1)
PA14_20230	2.20	4.58	nosR	Nitrous oxide reductase maturation protein NosR
PA14_04180	2.20	4.60	carO	calcium-regulated OB-fold protein CarO
PA14_20150	2.21	4.63	nosL	Nitrous oxide reductase maturation protein, outer-membrane lipoprotein NosL
PA14_70970	2.21	4.64	betI	Transcriptional regulator BetI, TetR family
PA14_63130	2.34	5.05	PA4775	hypothetical protein
PA14_52900	2.37	5.17		Acyl-CoA dehydrogenase STM0857
PA14_52880	2.39	5.23		2-methyl citrate dehydratase MmgE/prpD family
PA14_52910	2.39	5.26		COGs COG3777
PA14_52890	2.58	5.96		PA14_RS21535; Putative ring-cleaving dioxygenase; VOC family virulence protein
PA14_34190	2.75	6.73	msuD	FMNH2-dependent alkanesulfonate monooxygenase (EC 1.14.14.5)
PA14_29750	2.77	6.82		hypothetical protein
PA14_38395	3.05	8.30	mexX	Multidrug efflux system, membrane fusion component
PA14_34170	3.12	8.67		
PA14_38410	3.15	8.87	mexY	Multidrug efflux system, inner membrane proton/drug antiporter (RND type)
PA14_34180	3.22	9.30	msuE	FMN reductase (NADH) (EC 1.5.1.42)
PA14_63110	3.77	13.67	speD2	S-adenosylmethionine decarboxylase proenzyme (EC 4.1.1.50), prokaryotic class 1B

PA14_63120	3.92	15.15	speE2	spermidine synthetase
PA14_41280	5.75	53.99	PA1797	Beta-lactamase class C-like and penicillin binding proteins (PBPs) superfamily

FC, fold change compared to untreated control condition

Table S5 RNAseq transcriptome analysis of PA14 in response to G3KL exposure (0.25 x MIC)

PA14 locus	logFC	FC	gene	description
PA14_64620	-1.77	0.29		Flavodoxin reductases (ferredoxin-NADPH reductases) family 1
PA14_18790	-1.67	0.32	opmE	Multidrug efflux system, outer membrane factor lipoprotein
PA14_64610	-1.52	0.35		Linoleoyl-CoA desaturase (EC 1.14.19.3)
PA14_13750	-1.52	0.35	narK1	Nitrate/nitrite transporter NarK/U 1
PA14_18860	-1.41	0.38		SAM-dependent methyltransferase
PA14_18850	-1.37	0.39		Formerly called adenylosuccinate lyase
PA14_RS30730	-1.17	0.44		hypothetical protein
PA14_06830	-1.16	0.45	norB	Nitric-oxide reductase subunit B (EC 1.7.99.7)
PA14_13770	-1.16	0.45	narK2	Nitrate/nitrite transporter NarK/U
PA14_67340	-1.15	0.45		Putative transporter
PA14_19590	-1.12	0.46		Organosulfonate utilization protein SsuF
PA14_67350	-1.09	0.47	hutU	Urocanate hydratase (EC 4.2.1.49)
PA14_06810	-1.09	0.47	norC	Nitric-oxide reductase subunit C (EC 1.7.99.7)
PA14_53400	-1.06	0.48		Putative oxidoreductase
PA14_34740	-1.05	0.48		hypothetical protein
PA14_55750	-1.04	0.49		probable chemotaxis transducer
PA14_31500	-1.03	0.49		Acyl-CoA synthetases (AMP-forming)/AMP-acid ligases
PA14_22740	-1.00	0.50		P pilus assembly/Cpx signaling pathway
PA14_25080	1.00	2.01	fadB	Enoyl-CoA hydratase (EC 4.2.1.17)
PA14_25840	1.01	2.02		Electron transfer flavoprotein-ubiquinone oxidoreductase (EC 1.5.5.1)
PA14_68110	1.04	2.05	PA5157	Transcriptional regulator, MarR family
PA14_01720	1.05	2.08	ahpF	Alkyl hydroperoxide reductase protein F
PA14_63640	1.06	2.08	fadH2	2,4-dienoyl-CoA reductase [NADPH] (EC 1.3.1.34)
PA14_42090	1.12	2.18		3-ketoacyl-CoA thiolase (EC 2.3.1.16)
PA14_23520	1.17	2.24	PA3137	Inner-membrane proton/drug antiporter (MSF type) of tripartite multidrug efflux system
PA14_40850	1.23	2.34		Fructose-2,6-bisphosphatase
PA14_66530	1.23	2.35	PA5032	Transcriptional regulator, AraC family

PA14_66450	1.24	2.36		hypothetical protein
PA14_03590	1.27	2.41		hypothetical protein
PA14_04180	1.35	2.55	carO	calcium-regulated OB-fold protein CarO
PA14_21530	1.38	2.61		FOG: Ankyrin repeat
PA14_23530	1.47	2.76	PA3136	Membrane fusion component of MSF-type tripartite multidrug efflux system
PA14_06640	1.47	2.77		Acyl-CoA dehydrogenase 2 [fadN-fadA-fadE operon] (EC 1.3.8.7)
PA14_63110	1.56	2.95	speD2	S-adenosylmethionine decarboxylase proenzyme (EC 4.1.1.50), prokaryotic class 1B
PA14_18310	1.70	3.24	arnF	Undecaprenyl phosphate-aminoarabinose flippase subunit ArnF
PA14_49080	1.72	3.29	PA1187	Acyl-CoA dehydrogenase
PA14_18370	1.78	3.43	arnB	UDP-4-amino-4-deoxy-L-arabinose--oxoglutarate aminotransferase (EC 2.6.1.87)
PA14_18320	1.83	3.55	arnE	Undecaprenyl phosphate-aminoarabinose flippase subunit ArnE
PA14_18330	1.84	3.58	arnT	Undecaprenyl phosphate-alpha-4-amino-4-deoxy-L-arabinose arabinosyl transferase
PA14_53300	1.85	3.61	ahpB	Alkyl hydroperoxide reductase subunit C-like protein, antioxidant, oxyR regulated
PA14_63120	1.85	3.62	speE2	spermidine synthetase
PA14_18340	1.87	3.65	arnD	4-deoxy-4-formamido-L-arabinose-phosphoundecaprenol deformylase ArnD
PA14_18350	1.89	3.70	arnA	UDP-4-amino-4-deoxy-L-arabinose formyltransferase (EC 2.1.2.13)
PA14_18300	1.96	3.89	ugd	UDP-glucose 6-dehydrogenase (EC 1.1.1.22)
PA14_18360	2.13	4.39	arnC	Undecaprenyl-phosphate 4-deoxy-4-formamido-L-arabinose transferase (EC 2.4.2.53)
PA14_44311	2.40	5.28	cprA	Nucleoside-diphosphate-sugar epimerases (NADP bdg domain)
PA14_24360	3.20	9.16		autotransporter in OM, serine-type endopeptidase

FC, fold change compared to untreated control condition

Table S6 RNAseq transcriptome analysis of PA14 in response to T7 exposure (0.25 x MIC)

PA14 locus	logFC	FC	gene	description
PA14_19590	-1.85	0.28	ssuF	Organosulfonate utilization protein SsuF
PA14_02500	-1.69	0.31	exbB1	MotA/TolQ/ExbB proton channel family protein
PA14_13750	-1.63	0.32	narK1	Nitrate/nitrite transporter NarK/U 1
PA14_18790	-1.41	0.38	opmE	Multidrug efflux system, outer membrane factor lipoprotein
PA14_22320	-1.39	0.38		Uncharacterized membrane protein SO_4740
PA14_02510	-1.35	0.39	exbD1	Biopolymer transport protein ExbD/TolR
PA14_12970	-1.32	0.40	tauD	Alpha-ketoglutarate-dependent taurine dioxygenase (EC 1.14.11.17)
PA14_07980	-1.31	0.40		pyocin R2_PP, conserved hypothetical protein
PA14_07970	-1.28	0.41		Phage TraR/YbiI family protein
PA14_19560	-1.25	0.42	ssuD	FMNH2-dependent alkanesulfonate monooxygenase (EC 1.14.14.5)
PA14_21530	-1.23	0.43		FOG: Ankyrin repeat
PA14_22740	-1.21	0.43		P pilus assembly/Cpx signaling pathway, periplasmic inhibitor/zinc-resistance associated protein
PA14_02490	-1.20	0.43	tonB2	putative TonB-dependent receptor
PA14_61040	-1.17	0.44	katB	Catalase KatE (EC 1.11.1.6)
PA14_52480	-1.16	0.45		hypothetical protein
PA14_12940	-1.14	0.45	tauB	Taurine ABC transporter, ATP-binding protein TauB
PA14_19580	-1.13	0.46	ssuB	Alkanesulfonate ABC transporter ATP-binding protein SsuB
PA14_13770	-1.08	0.47	narK2	Nitrate/nitrite transporter NarK/U
PA14_52490	-1.08	0.47		hypothetical protein
PA14_34770	-1.06	0.48	tauA	Taurine ABC transporter, substrate-binding protein TauA
PA14_19570	-1.05	0.48	ssuC	Alkanesulfonate ABC transporter permease protein SsuC
PA14_12960	-1.03	0.49	tauC	Taurine ABC transporter, permease protein TauC
PA14_37990	-1.01	0.50		RNA polymerase ECF-type sigma factor
PA14_18860	-1.01	0.50		SAM-dependent methyltransferase
PA14_08210	-1.01	0.50		hypothetical protein
PA14_08230	-1.00	0.50		hypothetical protein
PA14_04790	1.01	2.02		FIG028932: hypothetical protein

PA14_04780	1.04	2.06		FIG022869: Oxidoreductase, GMC family
PA14_34290	1.09	2.13		Coenzyme F420-dependent N5,N10-methylene tetrahydromethanopterin reductase
PA14_41280	1.13	2.19	PA1797	Beta-lactamase class C-like and penicillin binding proteins (PBPs) superfamily
PA14_63160	1.17	2.25	pmrB	Sensory histidine kinase pmrB
PA14_63150	1.28	2.42	pmrA	Two-component system response regulator pmrA
PA14_RS04385	1.62	3.07		PA14_10890; NAD(P)-dependent oxidoreductase
PA14_63130	1.95	3.87	PA4775	hypothetical protein
PA14_04180	2.46	5.48	carO	calcium-regulated OB-fold protein CarO
PA14_63110	3.01	8.05	speD2	S-adenosylmethionine decarboxylase proenzyme (EC 4.1.1.50), prokaryotic class 1B
PA14_18370	3.02	8.10	arnB	UDP-4-amino-4-deoxy-L-arabinose--oxoglutarate aminotransferase (EC 2.6.1.87)
PA14_18320	3.25	9.53	arnE	Undecaprenyl phosphate-aminoarabinose flippase subunit ArnE
PA14_63120	3.28	9.73	speE2	spermidine synthetase
PA14_18350	3.32	10.00	arnA	UDP-4-amino-4-deoxy-L-arabinose formyltransferase (EC 2.1.2.13)
PA14_18310	3.34	10.12	arnF	Undecaprenyl phosphate-aminoarabinose flippase subunit ArnF
PA14_18330	3.37	10.35	arnT	Undecaprenyl phosphate-alpha-4-amino-4-deoxy-L-arabinose arabinosyl transferase
PA14_18360	3.37	10.36	arnC	Undecaprenyl-phosphate 4-deoxy-4-formamido-L-arabinose transferase (EC 2.4.2.53)
PA14_18340	3.47	11.10	arnD	4-deoxy-4-formamido-L-arabinose-phosphoundecaprenol deformylase ArnD (EC 3.5.1.n3)
PA14_18300	3.52	11.46	ugd	UDP-glucose 6-dehydrogenase (EC 1.1.1.22)
PA14_44311	3.93	15.28	cprA	Nucleoside-diphosphate-sugar epimerases
PA14_24360	4.85	28.80		autotransporter in OM, serine-type endopeptidase

FC, fold change compared to untreated control condition
